# Codelivery of DOX and siRNA by folate-biotin-quaternized starch nanoparticles for promoting synergistic suppression of human lung cancer cells

**DOI:** 10.1080/10717544.2019.1606363

**Published:** 2019-04-29

**Authors:** Liangping Li, Suoju He, Lizhen Yu, Ezzat H Elshazly, Hui Wang, Kuanmin Chen, Song Zhang, Lixia Ke, Renmin Gong

**Affiliations:** a College of Life Science, Anhui Normal University, Wuhu, P R China;; b Department of Physical Education, Anhui College of Traditional Chinese Medicine, Wuhu, P R China;; c School of Pharmacy, Wannan Medical College, Wuhu, P R China;; d Department of Botany and Microbiology, Faculty of Science, Al Azhar University, Assiut, Egypt

**Keywords:** Folate-biotin-quaternized starch nanoparticle, doxorubicin, siRNA, codelivery, human lung cancer cell lines

## Abstract

In this paper, the self-assembled folate-biotin-quaternized starch nanoparticles (FBqS NPs) were used as carrier system of doxorubicin (DOX) and siRNA^IGF1R^ for the codelivery of both into human lung adenocarcinoma cell lines (A549 cells) in vitro. The cytotoxicity, targeted ligand competition, cell proliferation inhibition, cellular uptake, endocytosis mechanism and target protein suppression of drug-loaded FBqS NPs were evaluated in detail. Compared with several other drug formulations under same condition, siRNA^IGF1R^/DOX/FBqS NPs exhibited the greatest cytotoxicity to A549 cells and the cytotoxicity was competitively inhibited by free folate in dose-dependent manner. The A549 cells treated by siRNA^IGF1R^/DOX/FBqS NPs showed the lowest cell proliferation capacity. The energy-dependent clathrin- and caveolae-mediated endocytosis might be the primary cellular uptake mechanism of drug-loaded FBqS NPs. The expression of IGF1R protein in A549 cells treated by siRNA^IGF1R^/FBqS NPs declined dramatically. So the FBqS NPs were expected as the co-carrier system of chemotherapeutants and siRNAs for future clinical application.

## Introduction

Cancer is an uncontrollable illness worldwide with high mortality rate. At present, the main tumor treatments, such as surgery, chemotherapy, and radiotherapy, still have some limitations which cripple the therapeutic effect. Surgery may damage adjacent healthy tissues and even cause metastasis of cancer cells. Radiotherapy always brings about some grievous side effects, such as osteoradionecrosis, anorexia, swallowing dysfunction, dyspnea and oral mucositis (Chulpanova et al., 2018; Hague et al., 2018; Hussein et al., 2018). Chemotherapy, the most common cancer treatment, is mainly performed through intravenous injection of small molecule anticancer drugs to suppress tumor cells. Unfortunately, the distribution of anticancer drugs in human body is nonspecific to tumor tissue, so both tumor tissue and normal tissue are damaged by chemotherapeutants (Li, Sun, et al., 2018). Besides, cancer cells are protected from apoptosis by multidrug-resistant (MDR), which also severely weakens the effects of chemotherapy (Suo et al., 2016; Zheng et al., 2016; Suo et al., 2017; Hou et al., 2018). As the first leading causes of cancer death in China, lung cancer has drawn great concern in recent years (Bica-Pop et al., 2018; Collett et al., 2018; Zhou et al., 2018). The above-untargeted drug distribution and MDR are also found in lung cancer chemotherapy, which may lead to low survival rate, high recurrence rate and even therapeutic failure in lung cancer treatment. So, it is very important and urgent to find out novel approaches to improve the therapeutic effect in lung cancer chemotherapy (Collett et al., 2018; Li, Zhang, et al., 2018; Zhou et al., 2018).

Small interfering RNAs (siRNAs) are the short double-stranded RNAs with sequence-specific gene-silencing function (Fernandes et al., 2012), which can be used to cause the degradation of target mRNA, suppress the expression of target protein and then induce the apoptosis of cells. The gene silencing technique of siRNAs has recently been utilized to treat some diseases, including cancer (Novo et al., 2014; Zheng et al., 2017). The siRNAs have been previously used to inhibit the expression of antiapoptotic proteins in tumor cells, including Survivin (Salzano et al., 2014; Wang et al., 2016), Bcl-2 (Chen et al., 2017; Suo et al., 2017), Cy5 (Gao et al., 2014; Sun et al., 2015), MDR1 (Tsubaki et al., 2012; Hu et al., 2014), P-gp (Suo et al., 2016; Xia et al., 2017) and so on. Insulin-like growth factor 1 receptor (IGF1R) is a transmembrane protein, which belongs to receptor family of tyrosine kinases and is implicated in several cancers including lung, breast and prostate cancers (Jones et al., 2004; Warshamana-Greene et al., 2005). In some cases, the antiapoptotic actions of IGF1R enable tumor cells to resist the cytotoxicity of chemotherapeutants or radiotherapy. So IGF1R can be regarded as one of target sites in cancer treatment (Hilmi et al., 2008; Dai & Tan, 2015; Ma et al., 2017; Zhao et al., 2017). Because naked siRNAs are rapidly degraded by RNAase in human body and negatively charged siRNAs can hardly penetrate cell membrane, the intracellular delivery of siRNAs urgently requires the safe and efficient carrier system (Fernandes et al., 2012; Guzman-Villanueva et al., 2014; Novo et al., 2015; Ahmadzada et al., 2018). Although the virus as vector of siRNAs has higher cell transfection efficiency, the safety still is the biggest obstacle to its clinical application (Zhu et al., 2010; Nuhn et al., 2012; Tekade et al., 2016; Xia et al., 2018). Recently, non-viral carriers have attracted more and more attention.

Starch, an agricultural product, has been widely used in the medical field including as drug delivery system (Chen et al., 2019; Massoumi et al., 2018), because of its natural characteristics such as biocompatibility, biodegradability, non-immunogenicity, non-toxicity and easy chemical modification. In our previous work (Li et al., 2017), the quaternized starch was used to fabricate the self-assembled folate-biotin-quaternized starch nanoparticles (FBqS NPs) as the co-carrier of siRNA and DOX. The physicochemical characteristics of FBqS NPs were characterized by TEM, DLS, ^1^H-NMR. The polydispersity index, critical aggregation concentration, drug loading content and encapsulation efficiency, serum stabilities, blood compatibility, drugs release curves of nanocarrier were evaluated in detail. The FBqS NPs had spherical core/shell structure with average diameter of 109 nm and positive charge (Z-potential: 28.59 ± 2.78 mV), which enabled them to effectively co-encapsulate hydrophobic anticancer drugs and negatively charged siRNAs. The FBqS NPs can effectively protect the encapsulated siRNA from degradation of RNAase in serum for a long time. The release behaviors of DOX and siRNA from FBqS NPs were all pH-responsive, and drugs were more liable to release in acidic environment. In this study, the FBqS NPs as encapsulation platform of DOX and siRNA^IGF1R^ for the co-delivery of both into A549 cells (human lung cancer cell lines) were completely evaluated. The cytotoxicity, targeted ligand competition, cell proliferation inhibition, cellular uptake, endocytosis mechanism and target protein suppression of drug-loaded FBqS NPs were estimated in detail. The aim of the present study is to provide new strategies for overcoming MDR associated with conventional anticancer drugs.

## Materials and methods

### Materials

DOX hydrochloride was purchased from Nanjing Oddfoni Biological Technology Co., Ltd (Nanjing, China). The siRNA^IGF1R^ with the sequence of 5′-CAUACUGCGCUCUAUAGAUTT-3′ was selected for targeting the IGF1R gene. The double-stranded siRNA^IGF1R^ and FAM-labeled siRNA^IGF1R^ (FAM: carboxyfluorescein) were designed and chemically synthesized by GenePharm Co., Ltd (Shanghai, China). 3-(4,5-Dimethylthiazol-2-yl)-2,5-diphenyltetrazolium bromide (MTT) cell proliferation and cytotoxicity assay kit, Annexin V-FITC apoptosis detection kit, Hoechst 33342, 4% paraformaldehyde, folate, biotin, RPMI-1640 medium, phosphate-buffered saline (PBS), fetal bovine serum (FBS), bicinchoninic acid (BCA) protein assay kit and polyvinylidene difluoride (PVDF) membrane were provided by Sangon Biotech Co., Ltd (Shanghai, China). Mouse monoclonal IGF1R beta chain antibody and HRP-conjugated beta-actin antibody, HRP-conjugated goat anti-mouse IgG (H + L) were from Proteintech Group, Inc (USA). All other reagents used were analytical grade and ultrapure water (18.25 MΩ) was used throughout the study.

### Cell lines and culture condition

The human A549 lung adenocarcinoma cell lines were used. The cells were routinely cultured in folate-free RPMI-1640 medium supplemented with 10% fetal bovine serum (FBS) and 1% penicillin–streptomycin in a humidified 5% CO_2_/95% atmosphere incubator at 37 °C. The cells in logarithmic growth phase were used and all experiments were repeatedly carried out at least three times on separate days.

### Cell viability

The MTT assay and flow cytometry (FCM) were used to assess the cytotoxicity of free DOX, blank and drug-loaded FBqS NPs against A549 cells. The preparation of the blank and various drug-loaded BqS and FBqS NPs in different formulations and drug concentrations could be found in our previous study (Li et al., 2017).

For MTT assay, A549 cells were seeded in 96-well culture plate (5 × 10^3^ cells/well) and incubated for 12 h, then culture medium was replaced with fresh culture medium containing blank FBqS NPs, free DOX, siRNA^IGF1R^/FBqS NPs, DOX/FBqS NPs or siRNA^IGF1R^/DOX/FBqS NPs at equivalent concentration of siRNA^IGF1R^, DOX and (or) FBqS NPs. The concentration ranges of siRNA^IGF1R^, DOX and FBqS NPs were from 0.7–4.2 mg/l, 2–12 mg/l and 26.7–160.2 mg/l, respectively. Every drug concentration was repeatedly conducted in five separate wells and with the cells without any treatment as control. After 48 h of incubation, the cells were washed twice with cold PBS, then 100 μl fresh culture medium containing MTT reagent (0.5 mg/ml) was added into each well and cells were incubated for another 4 h. The MTT reagent was removed and 100 μl DMSO was added into each well with continuously shaking for 10 min to dissolve the formazan crystals. Finally, the percentage of cell viability relative to the control group was measured at wavelength of 570 nm using a microplate reader (TECAN Spark, Switzerland).

For FCM, A549 cells were seeded in 6-well plate (5 × 10^5^ cells/well) and incubated for 12 h. Then the medium was replaced by fresh culture medium containing blank FBqS NPs, free DOX, siRNA^IGF1R^/FBqS NPs, DOX/FBqS NPs or siRNA^IGF1R^/DOX/FBqS NPs with constant concentration of 3.5 mg/l siRNA^IGF1R^, 10 mg/l DOX or (and) 133.3 mg/l FBqS NPs. The cells of control group were incubated in culture medium without any treatment. After 48 h of incubation, the cells were washed twice with cold PBS and harvested by trypsinization. The cells were washed again and centrifuged for removing supernatant. The suspension cells were stained by Annexin V-FITC and measured by FCM, the results were analyzed by FlowJo V10 software.

### Free folate competition

The previous studies confirmed that folate receptors were overexpressed on the membrane of A549 cells (Muthukumar et al., 2014; Piras et al., 2014; Hu et al., 2015; Kato et al., 2017; Singh et al., 2017; Tanino et al., 2018). In the current study, the folate competitive inhibition was determined by MTT assay and images of confocal laser scanning microscopy (CLSM).

For MTT assay, A549 cells (5 × 10^3^ cells/well) in 96-well plate were incubated for 12 h, then the culture medium was replaced with fresh culture medium containing varying concentration of free folate and constant concentration of siRNA^IGF1R^/DOX/FBqS NPs (siRNA^IGF1R^: 2.1 mg/l, DOX: 6 mg/l). The subsequent experimental procedures were the same as the MTT assay described in Section ‘Cell viability’.

For CLSM image, A549 cells were seeded in confocal dishes (diameter: 35 mm) at the density of 5 × 10^5^ cells/dish and incubated for 12 h. Then, the cells were exposed to the fresh culture medium containing a constant concentration of free DOX, FAM-siRNA^IGF1R^/DOX/BqS NPs or FAM-siRNA^IGF1R^/DOX/FBqS NPs (siRNA^IGF1R^: 0.9 mg/l, DOX: 2.5 mg/l) with or without free folate (1000 mg/l). After incubation for 8 h, the cells were washed, fixed, and then labeled nuclei with Hoechst 33342 (Wang et al., 2015). CLSM was used to visualize the uptake of FAM-siRNA^IGF1R^ (green fluorescence) and DOX (red fluorescence) by A549 cells.

### Wound healing assay

The wound healing assay was utilized to examine the inhibition of drugs to the proliferation and migration of A549 cells. Briefly, A549 cells were seeded in dishes (diameter: 35 mm) at the density of 5 × 10^5^ cells/dish and cultured for 12 hours to make cells attach and grow to the confluence of 80%. Then the linear scratch wound was made on the cell monolayer in dish with a 200 μl sterile pipette tip. The scratched cell monolayer was treated with culture medium containing constant concentration of free DOX, DOX/BqS NPs, DOX/FBqS NPs or siRNA^IGF1R^/DOX/FBqS NPs (siRNA^IGF1R^: 1.4 mg/l, DOX: 4 mg/l) with the sample without any drug treatment as control and incubated for 48 h. The samples were later washed triple with cold PBS and fixed with 4% paraformaldehyde. The change in wound width before and after treatment was observed with an inverted microscope (Olympus IX7, Japan). The wound width was measured by Image J software and the wound healing rate was calculated with the following equation:

$$\text{Wound healing rate}=\left({\text{W}}_{0}–\text{W}\right)/{\text{W}}_{0}$$

where W_0_ and W are the wound width of sample at 0 h and 48 h, respectively.

### Cellular uptake experiment

The cellular uptake and intracellular distribution of free DOX and FAM-siRNA^IGF1R^/DOX/FBqS NPs in A549 cells were observed and compared by CLSM (Olympus FV1000, Japan). Briefly, A549 cells were seeded in confocal dishes (5 × 10^5^ cells/dish) and incubated for 12 h. Then, the cells were incubated in the fresh culture medium containing constant concentration of free DOX or FAM-siRNA^IGF1R^/DOX/FBqS NPs (siRNA^IGF1R^: 0.9 mg/l, DOX: 2.5 mg/l) for different time intervals (1 h, 4 h, and 8 h). The cells were washed triple with cold PBS, fixed with 4% paraformaldehyde for 30 min, and then stained nuclei with Hoechst 33342. The CLSM images of FAM-siRNA^IGF1R^ (green), DOX (red) and Hoechst 33342 (blue) were observed for revealing the cellular uptake and internalization process of free DOX and FAM-siRNA^IGF1R^/DOX/FBqS NPs.

### Endocytosis inhibition test

The CLSM and FCM were utilized to investigate the endocytosis mechanism of FAM-siRNA^IGF1R^/DOX/FBqS NPs with different endocytosis inhibitors (Kuhn et al., 2014; Tian et al., 2014; Zhang et al., 2015).

For CLSM, A549 cells were seeded in confocal dishes (5 × 10^5^ cells/dish) and cultured for 12 h. Then the cells were pre-incubated for 1 h at 37 °C with the following specific endocytosis inhibitors: chlorpromazine (CPZ, 100 mg/l) for clathrin-mediated endocytosis inhibitor, indomethacin (INDO, 100 mg/l) for caveolae-mediated endocytosis inhibitor, quercetin (Qu, 100 mg/l) for caveolae- and clathrin-independent endocytosis inhibitor, sodium azide (NaN_3_, 20 mg/l) for energy inhibitor, colchicine (COLC, 100 mg/l) for microtubule-dependent macropinocytosis inhibitor and folate (FA, 1000 mg/l) for folate-receptor competitive inhibitor. Subsequently, the cells were incubated in the fresh culture medium containing a constant concentration of FAM-siRNA^IGF1R^/DOX/FBqS NPs (siRNA^IGF1R^: 0.9 mg/l, DOX: 2.5 mg/l) for another 8 h. The later experimental procedures were the same as the CLSM described in Section ‘Free folate competition’.

For FCM, the A549 cells (5 × 10^5^ cells/well) in 6-well plate were incubated for 12 h. Then cells were respectively treated with the above endocytosis inhibitors and then FAM-siRNA^IGF1R^/DOX/FBqS NPs. The cells treated only with FAM-siRNA^IGF1R^/DOX/FBqS NPs were used as positive control and the cells without any treatment as a negative control. Subsequently, the cells were washed, harvested, washed again and centrifuged for removing supernatant. The fluorescence intensities of FAM-siRNA^IGF1R^ and DOX in suspension cells were measured by FCM.

### Western blotting

The western blotting was utilized to assay the affection of siRNA^IGF1R^/FBqS NPs to expression of IGF1R protein in A549 cells. Briefly, A549 cells were seeded in dishes (diameter: 35 mm) at the density of 5 × 10^5^ cells/dish and incubated to cells attached on the bottom of dishes. Then the cells were cultured in 2 ml fresh culture medium containing two doses of siRNA^IGF1R^/FBqS NPs (siRNA^IGF1R^: 1.5 or 3 mg/l) for 48 h with untreated cells as control. Subsequently, the cells were harvested, washed and the cellular proteins were extracted with ice-cold lysis buffer (Tris: 50 mM, pH = 7.4, NaCl: 150 mM, NP-40: 1%, SDS: 1%). The protein concentrations were measured using BCA protein assay kit. The equal amount of protein was separated on 8% sodium dodecyl sulfate-polyacrylamide gel electrophoresis (SDS-PAGE) and then transferred to PVDF membrane. The membrane was blocked and incubated overnight at 4 °C with mouse monoclonal anti-IGF1R antibodies. After washing the membrane with TBST solution, the membrane was again incubated with HRP-conjugated secondary antibody for 1 h. The protein bands were visualized with the enhanced chemiluminescence (ECL) detection system with β-actin as loading control. The image of protein bands was obtained using Chemiluminescent Imaging System (Tanon 5200, Shanghai).

### Statistical analysis

The two-tailed student’s *t*-test and One-Way ANOVA were used to determine the statistical differences between samples, the results of statistical analysis were shown as mean ± standard deviation. All statistical analyses were conducted by IBM SPSS statistic software. Statistical significance was judged at *p* < .05 (95% confidence interval).

## Results

### Cytotoxicity study

**Figure 1.**
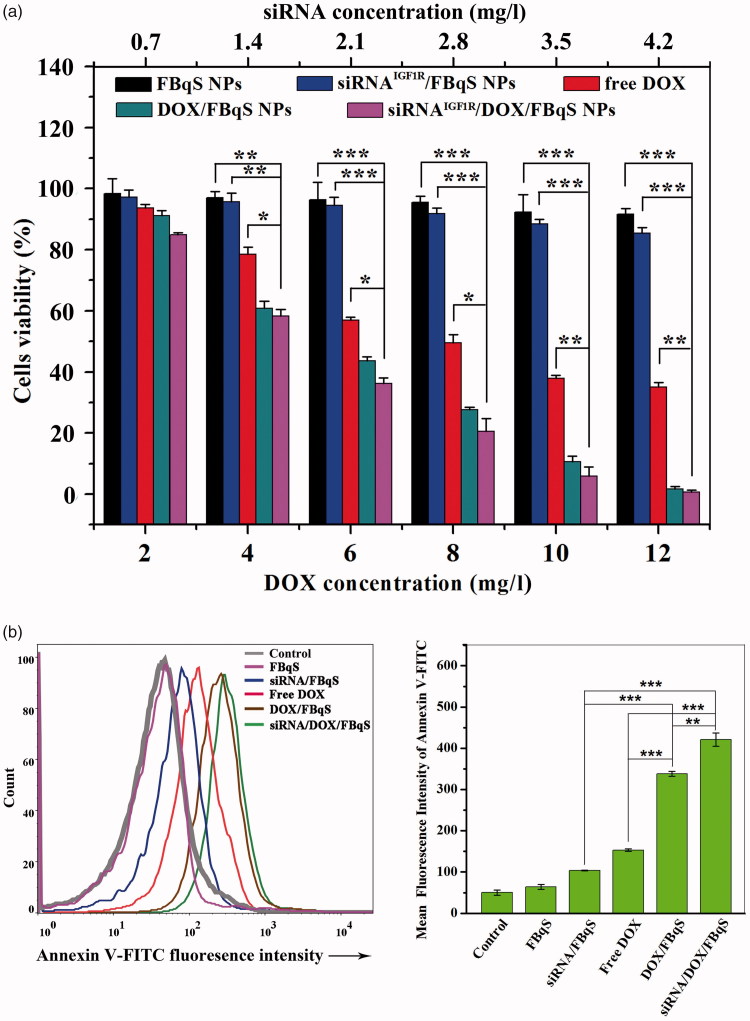
Cytotoxicity of blank FBqS NPs, free DOX, siRNA^IGF1R^/FBqS NPs, DOX/FBqS NPs and siRNA^IGF1R^/DOX/FBqS NPs against A549 cells by MTT (a) and FCM (b). FBqS NPs concentrations: 26.7 mg/l, 53.4 mg/l, 80.1 mg/l, 106.8 mg/l, 133.5 mg/l and 160.2 mg/l, corresponding to DOX concentration from 2 to 12 mg/l, respectively. Means ± SD (*n* = 5). *p < .05, **p < .01, ***p < .001.

The cytotoxicity of free DOX, blank and drug-loaded NPs against A549 cells was measured and evaluated by MTT and FCM assays. The result of MTT assay at various drug concentrations was shown in Figure 1(a). The cytotoxicity of blank FBqS NPs against A549 cells was negligible. Except for the group of siRNA^IGF1R^/FBqS NPs, the cytotoxicity of other administration groups enhanced as DOX concentration increased, showing the cytotoxicity was a function of DOX dose. The values of half inhibitory concentration (IC_50_) of free DOX, DOX/FBqS NPs and siRNA^IGF1R^/DOX/FBqS NPs were about 7.85, 5.95 and 5.21 mg/l, respectively. Because the siRNA^IGF1R^/FBqS NPs could not kill A549 cells directly, its IC_50_ value was hardly determined even in very high concentrations. The cytotoxicity of DOX/FBqS NPs was stronger than that of free DOX, which was due to folate-receptor-mediated endocytosis resulting in increased cellular drug uptake. The siRNA^IGF1R^/DOX/FBqS NPs was observed to have the highest cytotoxicity to A549 cells in four administration groups, which exhibited the synergistic inhibition effect of chemotherapeutics and siRNA to cancer cells. This ideal cytotoxic effect made the possible clinical application in the future for siRNA/DOX/FBqS NPs.

The apoptosis of A549 cells induced by free DOX, blank and drug-loaded NPs at equivalent drug concentration was also analyzed by FCM. The cell apoptosis was determined based on the fluorescence intensity of Annexin V-FITC in samples. As shown in Figure 1(b), the order of fluorescence intensity from strong to weak was siRNA^IGF1R^/DOX/FBqS NPs > DOX/FBqS NPs > free DOX > siRNA^IGF1R^/FBqS NPs > blank FBqS NPs = control. The cells incubated with siRNA^IGF1R^/DOX/FBqS NPs exhibited the strongest fluorescence intensity in all samples, which meant that the codelivery of DOX and siRNA^IGF1R^ by FBqS NPs had best apoptosis effect to A549 cells. The cells incubated with blank FBqS NPs exhibited close equal fluorescence intensity to the control, which illustrated that the cytotoxicity of blank FBqS NPs was neglectable. These results were highly consistent with those obtained by MTT assay.

### Folate competitive inhibition assay

**Figure 2.**
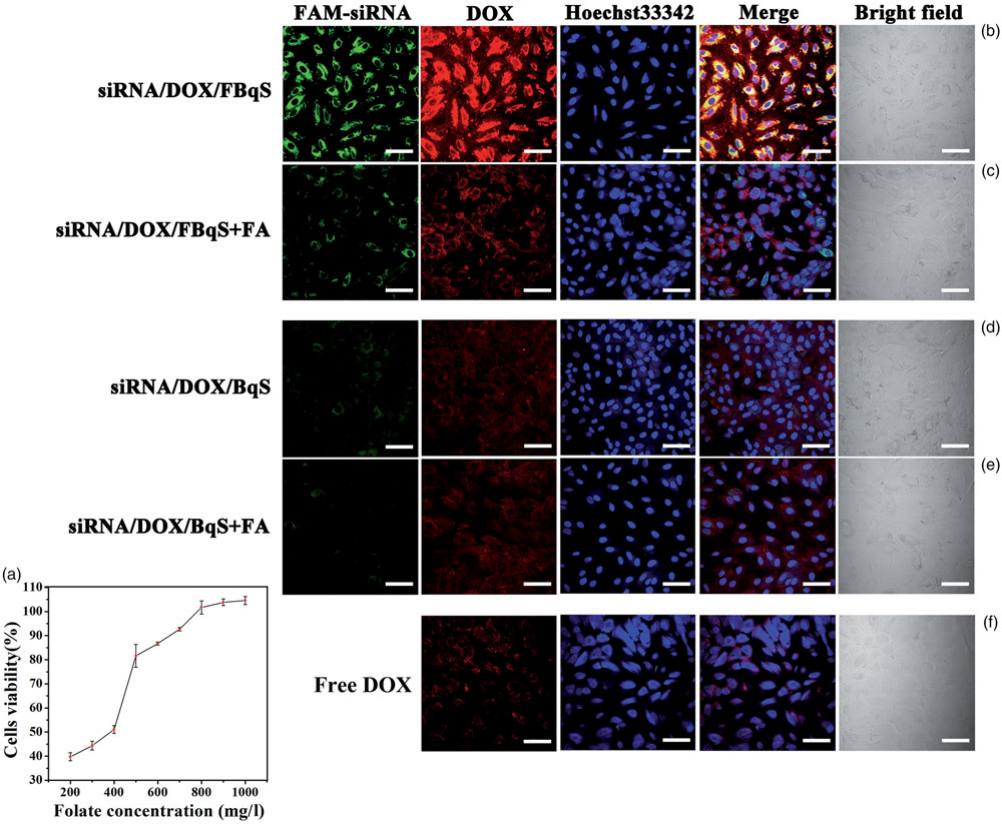
Viability of A549 cells incubated in culture medium containing constant concentration of siRNA^IGF1R^/DOX/FBqS NPs and varying concentration of free folate (a); Cellular uptake of FAM-siRNA^IGF1R^/DOX/FBqS NPs (b,c), FAM-siRNA^IGF1R^/DOX/BqS NPs (d,e) and free DOX (f) in A549 cells at constant concentration of siRNA^IGF1R^ and DOX without or with excess free folate. Scale bar, 60 μm.

Folate competition assay was conducted for further demonstrating the role of free folate in target delivery of siRNA^IGF1R^/DOX/FBqS NPs into A549 cells. The result in Figure 2(a) showed that cell viability increased with increasing free folate concentration in culture medium. For instance, the cell viability was only 39.4% in culture medium without free folate, but it increased to 92.7% in culture medium containing 700 mg/l of free folate. The above result hinted that free folate molecules inhibited the cellular uptake of siRNA^IGF1R^/DOX/FBqS NPs by competitive combination with the folate receptors on the A549 cell surface. The inhibitory effect was correlated with the folate concentration in culture medium.

The competitive inhibition effect of free folate in the cellular uptake of FAM-siRNA^IGF1R^/DOX/BqS NPs and FAM-siRNA^IGF1R^/DOX/FBqS NPs was also inspected by the images of CLSM. The fluorescence of FAM-siRNA^IGF1R^ (green) and DOX (red) in A549 cells displayed clearly the cellular uptake of FAM-siRNA^IGF1R^/DOX/BqS (FBqS) NPs. The cellular uptake of FAM-siRNA^IGF1R^/DOX/FBqS NPs was obviously reduced when the excess free folate (1000 mg/l) was added in culture medium (Figure 2(b,c)). Because of the lack of folate ligand on the BqS nanocarrier, the cellular uptake of FAM-siRNA^IGF1R^/DOX/BqS NPs was significantly less than that of FAM-siRNA^IGF1R^/DOX/FBqS NPs (Figure 2(b,d)) and there was little difference in cellular uptake of FAM-siRNA^IGF1R^/DOX/BqS NPs regardless of with or without excess free folate in culture medium (Figure 2(d,e)). In all administration groups, the cellular uptake of free DOX by A549 cells was least (Figure 2(f)). These results distinctly illustrated that the cellular internalization of siRNA and DOX was obviously enhanced by decorating folate ligand on nanocarrier, and the cellular uptake of drug-loaded FBqS NPs could be inhibited by free folate in culture medium. The enhanced cellular uptake and accumulation of FAM-siRNA^IGF1R^/DOX/FBqS NPs maybe relate to the folate-receptor-mediated endocytosis mechanism.

### Inhibition of cell migration

**Figure 3.**
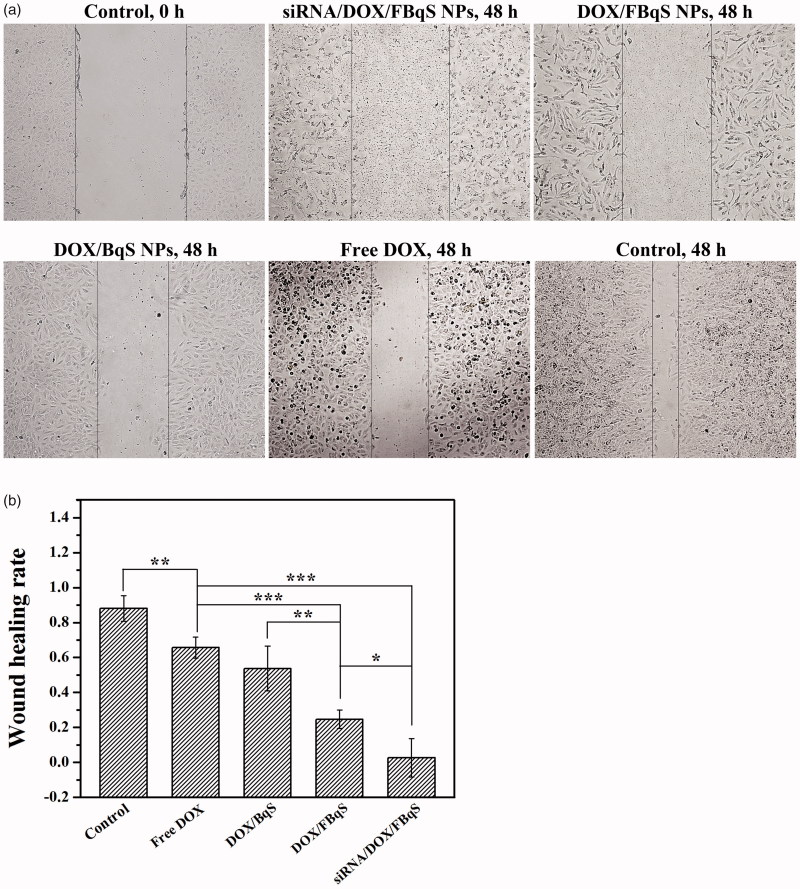
Wound healing assay of free DOX, DOX/BqS NPs, DOX/FBqS NPs and siRNA^IGF1R^/DOX/FBqS NPs in A549 cells (a); Wound healing rate of various drug formulations (b). Means ± SD (*n* = 3). *p < .05, **p < .01, ***p < .001.

The wound healing assay was utilized to evaluate the effect of free DOX and drug-loaded NPs on the proliferation and migration of A549 cells (Figure 3(a,b)). After incubation for 48 h, the siRNA^IGF1R^/DOX/FBqS NPs exhibited most effective inhibition to the migration of A549 cells with wound-healing rate of only 0.03. The wound-healing rate of A549 cell monolayer brought by DOX/FBqS NPs was 0.25, which was higher than that caused by siRNA^IGF1R^/DOX/FBqS NPs but lower than that (0.54) induced by DOX/BqS NPs. The highest wound-healing rate (0.66) was caused by free DOX in all administration groups. The wounded area on A549 cell monolayer, that had not received any treatment, became narrow and left only a small gap. Compared with free DOX, DOX/BqS NPs and DOX/FBqS NPs, the siRNA^IGF1R^/DOX/FBqS NPs exhibited the strongest inhibition effect to both proliferation and migration of A549 cells, which reflected the synergistic repression efficacy of siRNA and DOX to tumor cells.

### Cellular uptake pattern and intracellular distribution

**Figure 4.**
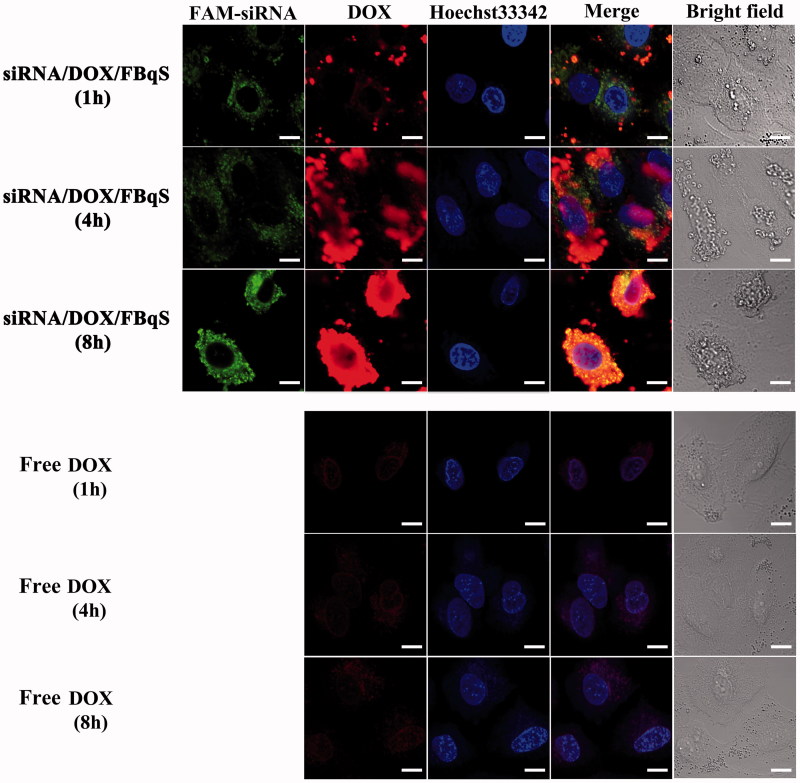
CLSM images of A549 cells incubated with FAM-siRNA^IGF1R^/DOX/FBqS NPs or free DOX at different time intervals. Scale bar, 10 μm.

The images of CLSM were used to investigate the cellular uptake of free DOX and FAM-siRNA^IGF1R^/DOX/FBqS NPs and the intracellular distribution of drugs in A549 cells (Figure 4). For the cells incubated with FAM-siRNA^IGF1R^/DOX/FBqS NPs for 1 h, DOX and FAM-siRNA^IGF1R^ had entered into A549 cells. It could be found that the FAM-siRNA^IGF1R^ presented uniform distribution in cytoplasm but the DOX distributed as scattered dots. After incubation of 4 h, the plenty of DOX and FAM-siRNA^IGF1R^ can be observed in cytoplasm and the small amount of DOX had entered into cell nucleus at the same time. After incubation for 8 hours, the DOX had covered the whole cell nucleus and FAM-siRNA^IGF1R^ further dispersed throughout the cytoplasm. But for A549 cells incubated with free DOX, only faint red fluorescence of DOX could be observed in cytoplasm and cell nucleus at any equal time intervals, compared with the group of FAM-siRNA^IGF1R^/DOX/FBqS NPs. This result clearly indicated the involvement of folate receptor-mediated endocytosis in the cellular uptake of FAM-siRNA^IGF1R^/DOX/FBqS NPs. Compared with the same drug concentration of free DOX, the FAM-siRNA^IGF1R^/DOX/FBqS NPs were easier to gather on the folate receptors anchored on membrane of A549 cells and so enhanced the cellular uptake of drugs.

### Cellular uptake mechanism

**Figure 5.**
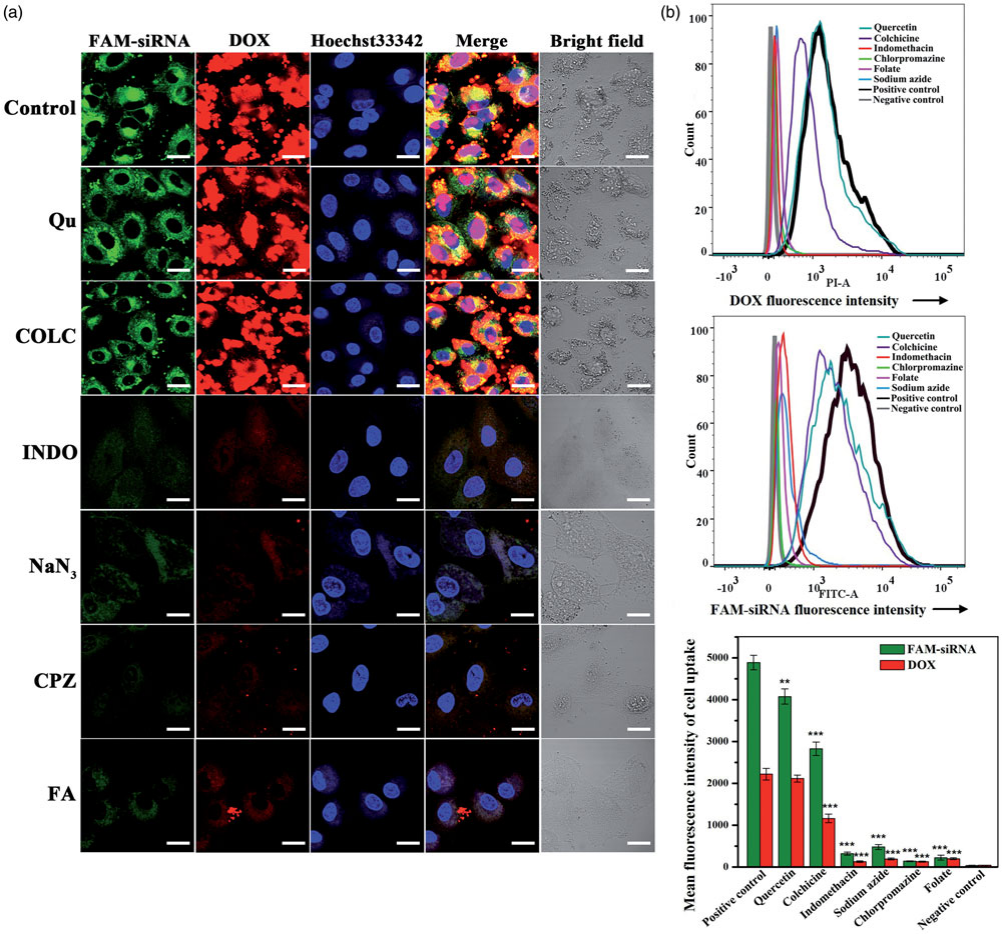
CLSM images of A549 cells incubated with FAM-siRNA^IGF1R^/DOX/FBqS NPs for 8 h in the presence of different endocytosis inhibitors (a); Quantitative fluorescence intensities of DOX and FAM-siRNA^IGF1R^ in A549 cells incubated with FAM-siRNA^IGF1R^/DOX/FBqS NPs and different endocytosis inhibitors measured by FCM (b). Means ± SD (*n* = 3). **p < .01, ***p < .001, compared with positive control. Scale bar, 20 μm.

The cellular uptake mechanism of FAM-siRNA^IGF1R^/DOX/FBqS NPs in A549 cells was studied by CLSM image. The specific endocytosis inhibitors were applied to block the corresponding transport pathway. The inhibition results of cellular uptake were shown in Figure 5(a). Compared with the control without inhibitor, Qu and COLC displayed little or no inhibition effect to the cellular uptake of drug-loaded NPs. On the contrary, the cellular uptake of drug-loaded FBqS NPs was significantly inhibited by INDO, NaN_3,_ and CPZ. The results distinctly indicated that the energy-dependent clathrin- and caveolae-mediated endocytosis were the main transport pathway of drug-loaded FBqS NPs taken into A549 cells. The intense cellular uptake suppression caused by free folate was highly consistent with the result of folate competition assay.

The endocytosis mechanism of drug-loaded FBqS NPs was further investigated by FCM (Figure 5(b)). The stronger fluorescence of DOX and FAM-siRNA were detected in the A549 cells incubated with Qu or COLC than those fluorescences exhibited in the cells incubated with other three endocytosis inhibitors (INDO, NaN_3_, and CPZ), which illustrated that INDO, NaN_3,_ and CPZ were effective endocytosis inhibitors of drug-loaded FBqS NPs but Qu and COLC exhibited only small influence on the cellular uptake of drug-loaded nanocarriers. This result was basically consistent with the conclusion drawn by CLSM images and the inhibitory effect of free folate was also similar to that of CLSM images.

### Suppression of target protein

**Figure 6.**
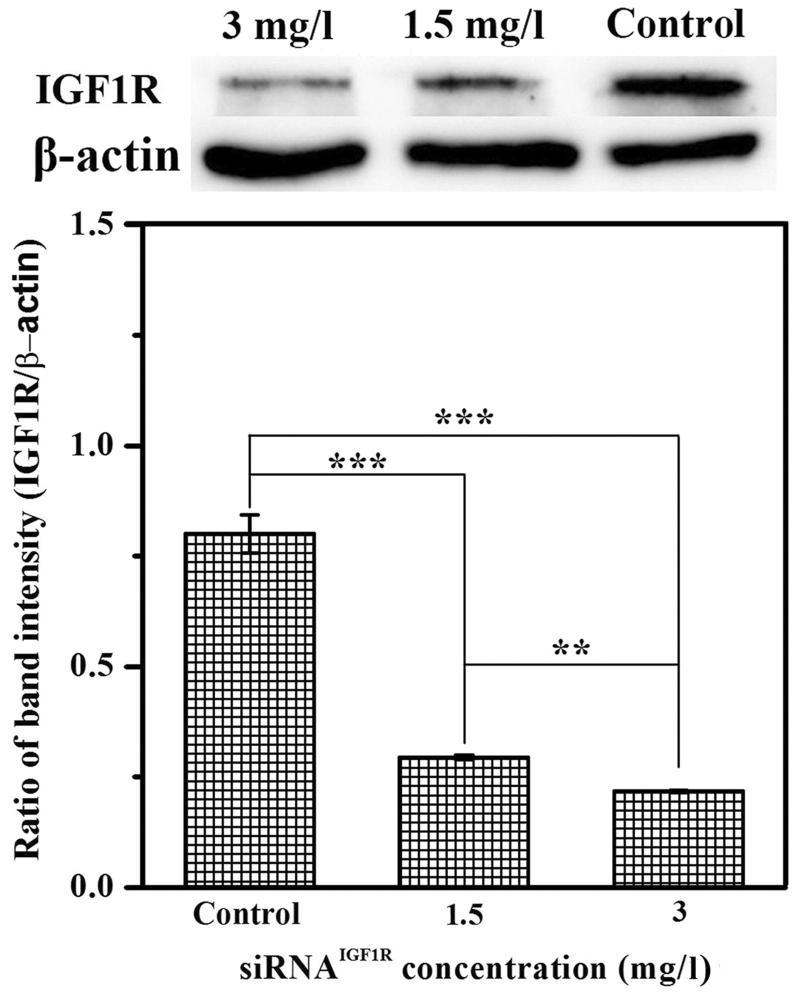
Downregulation of IGF1R protein in A549 cells after treatment with siRNA^IGF1R^/FBqS NPs, β-actin used as the loading control. Means ± SD (*n* = 3). **p < .01, ***p < .001.

The western blotting was utilized to assess the expression of IGF1R protein in A549 cells. As shown in Figure 6, the IGF1R protein level in A549 cells was reduced in a dose-dependent manner after treatment with siRNA^IGF1R^/FBqS NPs. Compared with the untreated cells, the ratio of IGF1R/β-actin decreased from 0.800 to 0.294 in the cells treated with siRNA^IGF1R^/FBqS NPs (siRNA^IGF1R^: 1.5 mg/l) and this ratio of proteins even dropped to 0.218 in the cells incubated with another siRNA^IGF1R^/FBqS NPs (siRNA^IGF1R^: 3 mg/l). This result suggested that siRNA delivered by FBqS NPs was able to be released into the cytoplasm and further brought the downregulation of target protein expression.

## Discussion

In this study, the self-assembled FBqS NPs serving as effective carrier system simultaneously delivered siRNA and DOX into A549 cells for combined cancer therapy. The drug-loaded FBqS NPs exhibited higher drug targeting delivery efficiency and good tumor suppression effect. The siRNA^IGF1R^/DOX/FBqS NPs showed the highest cytotoxicity and proliferation inhibition to A549 cells among several different drug formulations. The cellular uptake inhibition of siRNA^IGF1R^/DOX/FBqS NPs by free folate indicated that the drug-loaded FBqS NPs could specifically target folate-receptor-overexpressing cancer cells. It was surmised the energy-dependent clathrin- and caveolae-mediated endocytosis might be the principal cellular uptake mechanism of drug-loaded FBqS NPs. The expression of IGF1R protein in A549 cells was significantly suppressed by siRNA^IGF1R^/FBqS NPs. Therefore, the FBqS NPs were expected as potential co-carrier system of hydrophobic anticancer drug and siRNA for future clinical tumor therapy.
